# Ti_3_C_2_ MXenes-based catalysts for the process of α-pinene isomerization

**DOI:** 10.1039/d3ra05055f

**Published:** 2023-10-16

**Authors:** Bartosz Środa, Anna G. Dymerska, Piotr Miądlicki, Agnieszka Wróblewska, Beata Zielińska

**Affiliations:** a Department of Nanomaterials Physicochemistry, Faculty of Chemical Technology and Engineering, West Pomeranian University of Technology in Szczecin Piastów Ave. 42 71-065 Szczecin Poland bartosz.sroda@zut.edu.pl; b Department of Catalytic and Sorbent Materials Engineering, Faculty of Chemical Technology and Engineering, West Pomeranian University of Technology in Szczecin Piastów Ave. 42 71-065 Szczecin Poland

## Abstract

In this study, the catalytic performance of Ti_3_C_2_ MXene materials in the reaction of α-pinene isomerization was demonstrated. The influence of etching agents (HF, HF/H_2_SO_4,_ and HF/HCl; weight ratios of mixed acids: 1 : 3, 1 : 4, and 1 : 5) on removing Al atoms from MAX phase, creation of an accordion-like structure typical for MXenes and catalytic activity of the produced samples have been revealed. The MXene HF obtained by MAX phase HF treatment exhibited the highest activity (conversion of α-pinene 74.65 mol%), while materials produced with the mixed acids (HF/H_2_SO_4_ and HF/HCl) showed a significant reduction in the conversion of α-pinene (on average about 28-fold). However, these last samples displayed an increase of about 10 mol% in the selectivity to the most desirable product-camphene. The high activity of MXene HF is a result of a high concentration of acid sites (11.62 mmol g^−1^) – the concentration of acid sites in the samples obtained by MAX phase mixed acids treatment was about 2.5–5.5 times smaller. This work proposes possible mechanisms for the α-pinene isomerization reaction on the MXene HF and on the MXene HF/H_2_SO_4_*X* : *Y* and MXene HF/HCl *X* : *Y* in connection with their structure.

## Abstract 2

This study explores Ti__3__C__2__ MXene catalysts for the reaction of α-pinene isomerization to get highly valuable products such as camphene, limonene, and tricyclene.

## Graphical abstract

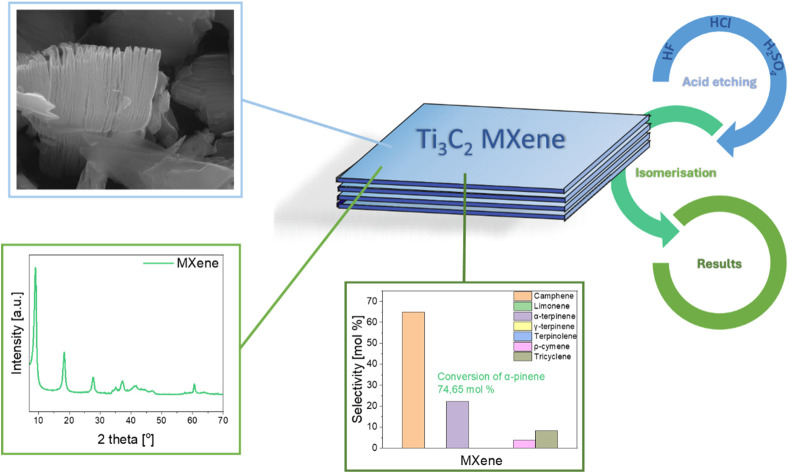

## 1. Introduction

After the discovery of graphene in 2004, scientists tried to find other two-dimensional (2D) materials. In 2011, a novel class of 2D materials termed MXenes attracted great interest from researchers due to their outstanding properties and range of potential applications. The general equation of MXene is M_*n*+1_X_*n*_T_*x*_, where M is an early transmission metal, X is carbon and/or nitrogen, *n* = 1, 2, 3 and T_*x*_ is a surface functional group (–F, =O, –OH). One of the most popular MXenes is titanium(iv) carbide (Ti_3_C_2_T_*x*_), which resembles layers in its structure.^1^ Ti_3_C_2_T_*x*_ has been tested in multiple application places. For instance, they can be tested as microwave-absorbing materials due to their positive dielectric loss ability. Their excellent flexibility and large surface area make them great candidates mainly for catalytic processes and supercapacitors. Moreover, MXenes are potential candidates as electrocatalysts in hydrogen evolution reactions or water splitting. However, in conventional heterogeneous catalysis used in organic chemistry, MXene materials are currently barely investigated.^2^

One of the directions of application of MXenes in heterogeneous catalysis may be the isomerization of α-pinene – the terpene compound of natural origin. Three main isomers of pinene occurring in nature were described: α-, β- and δ-pinene (each structural isomer has 2 enantiomeric forms (±)). Among these 3 isomers, α-pinene is the most abundant in nature. It has the smell of pine and is characterized by a fresh, forest aroma. The main source of α-pinene is turpentine, which is obtained from the resin of coniferous trees, mainly pine, by the steam distillation or gasoline extraction of pine stumps. α-Pinene is also found in essential oils obtained from pine, rosemary, cumin, thyme, basil, eucalyptus, and orange peels.^3–5^ α-Pinene is of great interest to scientists due to its biologically active properties (it is used as a fragrance in cosmetics, and in medicine for the treatment of respiratory infections or dissolution of kidney stones), as well as due to the possibility of obtaining derivatives of this terpene compound which also have numerous applications, including applications in medicine. The great advantage of α-pinene as the raw material for organic syntheses is that it is a relatively easily available and cheap raw material, and above all reproducible. This means that the syntheses carried out with the participation of α-pinene meet the principles of green chemistry and sustainable development. In addition, the method of isomerization of α-pinene proposed in this work does not require the use of a solvent, which is also beneficial for the environment and reduces the cost of this process.

The main α-pinene derivatives that are obtained in the isomerization process are camphene and limonene. Camphene is applied in the syntheses of many chemicals, for example, toxaphene (insecticide),^6^ and synthetic camphor, which is an important fragrance,^7^ and moreover, the reaction of camphene with guaiacol leads to valuable cyclohexanol products.^8^ We should also mention camphene applications in medicine, e.g. in the treatment of cancer.^9^ Limonene also found numerous applications in medicine, cosmetics, and organic synthesis. For example, the mixture of limonene, α-pinene, and 1,8-cineole shows a therapeutic activity similar to the standard antibiotics used in the treatment of respiratory diseases.^10^ The disproportionation reaction of limonene causes the formation of a mixture of p-cymene and *p*-menthane.^11^ Limonene is also converted to (−)-carvone, which is used as the peppermint flavor.^12^ Research is being conducted on the use of limonene as an additive for the production of plastics that will have contact with food (polyethylene, polystyrene, and polylactic acid).^13^ Other compounds which are formed in smaller amounts in the process of α-pinene isomerization (terpinolene, α-terpinene, γ-terpinene, and *p*-cymene) are commercially important compounds widely used in perfume, food, and organic industries and also in medicine.^14^

In the industrial isomerization of α-pinene, titanium oxide (TiO_2_) is used as the catalyst. The process is carried out at temperatures above 155 °C for 24 hours. The main products in this process are obtained: camphene, limonene, and tricyclene (the total selectivity of these compounds amounts to 70%).^15^ In addition, very good results present the work of Arata *et al.* They used TiO_2_ as a catalyst in the α-pinene isomerization process and achieved the conversion of this terpene compound amounted to 93%, at the temperature of 100 °C and for the reaction time of 2 hours but the main product of this process was *trans*-pinocarveol and its selectivity was 80%.^16^ The J. E. Sanchez-Velandia *et al.* obtained NT-TiO_2_-wc catalyst. NT-TiO_2_-wc was characterized by the conversion of α-pinene 99% and the selectivity of camphene 67%, for the reaction time amounted to 45 minutes.^17^ A major problem associated with the implementation of the process of isomerization of α-pinene on the industrial scale is the deactivation of the heterogeneous catalyst and the long reaction time. Therefore, the search for catalysts that would be characterized by a higher activity was started, which would shorten the reaction time, and at the same time, their use may allow camphene to be obtained with greater selectivity. These new catalysts should be catalysts that can be used many times in the isomerization process and also these materials should be easily regenerated – examples of such catalysts may be zeolite catalysts (natural origin or synthesized in a laboratory) or materials with a structure similar to the structure of zeolites, which are durable and relatively easy to regenerate. Studies related to the search for new, active catalysts for the α-pinene isomerization process were carried out, among others, with the following heterogeneous catalysts: Ga-SBA-15,^18^ PW/SBA-15,^19^ Ga-MCM-41,^20^ SO_4_/ZrO_4_/HMS,^21^ ferrite-type zeolites,^22^ acid treated natural zeolites,^23^ TCA/Y-zeolite,^24^ heat-treated natural zeolites^25^ natural clays.^26^ Very good results were obtained by conducting the studies on the natural zeolite–clinoptilolite, which was modified by washing with sulfuric acid solutions of appropriate concentrations (0.01–2 M). In this case, the conducting of the process for a very short time – 4 minutes and at the temperature of 70 °C, allowed for the complete conversion of α-pinene, and the selectivity of camphene and limonene amounted to 50 mol% and 30 mol%, respectively.^27^

In our previous study,^28^ multilayered and exfoliated Ti_3_C_2_ MXenes are proposed as a new class of catalysts for the reaction of α-pinene isomerization. Here, 100 mol% conversion of α-pinene in 7 h of process and the selectivity resulting in ∼60 mol% toward camphene formation was revealed. For that time, it was the highest selectivity and conversion reported in the current state of the art. Such promising results motivated us to continue research on Ti_3_C_2_T_*x*_ MXenes as catalysts in conventional heterogeneous catalysis, especially in the process of α-pinene isomerization. In the present work, Ti_3_C_2_T_*x*_ MXenes were synthesized by removing aluminum from MAX phase by etching in a mineral acids mixture (HF/HCl and HF/H_2_SO_4_). Here, the influence of HF/HCl and HF/H_2_SO_4_ weight ratios (1 : 3, 1 : 4, and 1 : 5) on the physicochemical properties and catalytic activity in the isomerization of α-pinene of the produced samples was studied. The obtained results were compared to the sample synthesized by etching Al from MAX phase with 48% hydrofluoric acid.

## 2. Experimental section

### 2.1. Materials

All chemicals used in this work: MAX phase (titanium aluminum carbide, Ti_3_AlC_2_, 99% purity, American Elements), hydrofluoric acid (HF, 48% concentration, Sigma-Aldrich), hydrochloric acid (HCl, 35–38% concentration, Sigma-Aldrich), sulfuric acid(vi) (H_2_SO_4_, 98% concentration, Sigma-Aldrich), ethanol (EtOH, 96% purity, Sigma-Aldrich) and α-pinene (98%, Sigma-Aldrich) were used as received, without further purification.

### 2.2. Synthesis of multilayered Ti_3_C_2_T_*x*_ MXenes

Multilayered Ti_3_C_2_T_*x*_ MXenes were produced *via* acidic etching aluminum from MAX phase (Ti_3_C_2_–Al–Ti_3_C_2_–Al–Ti_3_C_2_). Here, two different etching agents, HF/HCl and HF/H_2_SO_4_ with different weight ratios (1 : 3, 1 : 4, and 1 : 5), were used for removing Al atoms from MAX phase. The details of the etching solutions composition and MAX phase quantity are presented in Table 1. Briefly, MAX phase was carefully added to an appropriate mixture of acids and stirred for 24 h at room temperature (RT). After that, the obtained mixture was diluted with deionized water, and the precipitate was separated *via* centrifugation. The obtained product was washed with deionized water until the pH was ∼7. Finally, the received sample was dried overnight at 50 °C and stored at 5 °C for further analysis.^28^ The products were referred to as MXene HF/HCl *X* : *Y* and MXene HF/H_2_SO_4_*X* : *Y*, where *X* : *Y* means the acids weight ratios. Moreover, a reference sample of Ti_3_C_2_T_*x*_ MXene was also synthesized according to the same procedure as described above but using 48% HF for etching of Al form Ti_3_AlC_2_ (see Table 1). The sample was labeled as MXene HF.

**Table 1:** Composition of the etching solutions and MAX phase quantity for MXenes synthesis

Sample	HF [mL]	HCl [mL]	H_2_SO_4_ [mL]	MAX phase [mg]	Quantity of product [mg]
MXene HF	10	—	—	590	383
MXene HF/HCl 1 : 3	2	5.68	—	453	312
MXene HF/HCl 1 : 4	2	7.70	—	572	391
MXene HF/HCl 1 : 5	2	9.48	—	677	484
MXene HF/H_2_SO_4_ 1 : 3	2	—	3.70	336	247
MXene HF/H_2_SO_4_ 1 : 4	2	—	4.92	408	295
MXene HF/H_2_SO_4_ 1 : 5	2	—	6.16	481	388

### 2.3. Characterization

The morphology of obtained MXenes was characterized *via* scanning electron microscopy (VEGA3 Tescan). The crystallographic structure of samples was examined *via* X-ray diffraction (XRD), performed using an AERIS PANalytical X-ray diffractometer with Cu-Kα radiation. Raman spectroscopy (inVia Renishaw; *λ* = 785 nm) was used for the MXene vibrational signal. Thermogravimetric analysis (TGA) was performed in the temperature range between RT and 1400 °C, with a 10 °C min^−1^ heating ramp and 100 cm^−3^ min^−1^ airflow on an SDT Q600 thermal analyzer. To determine the specific surface area (SSA) and pore size distribution, we utilized the Brunauer–Emmett–Teller (BET) method, which involved conducting N_2_ sorption measurements following a preliminary sample degassing process using the Micrometrics ASAP 2460 system. The X-ray spectroscopy (XPS) measurements were conducted using Mg Kα (*hν* = 1253.6 eV) radiation in a Prevac (Poland) system equipped with a Scienta SES 2002 (Sweden) electron energy analyzer operating with constant transmission energy (*E*_p_ = 50 eV). The analysis chamber was evacuated to a pressure below 5 × 10^−9^ mbar.

The acid site concentration (As) of the samples was measured using the procedure described by Vilcocq *et al.*^29^ Firstly, 20 mg of the appropriate sample was added into 10 cm^3^ of 0.01 M NaOH solution and mixed at RT for 4 h. Next, the dispersion was filtered off and the pH of the filtrate was determined by titration using 0.01 M HCl in the presence of phenolphthalein as an indicator. The acid sites concentration was calculated from the formula (1) below:1
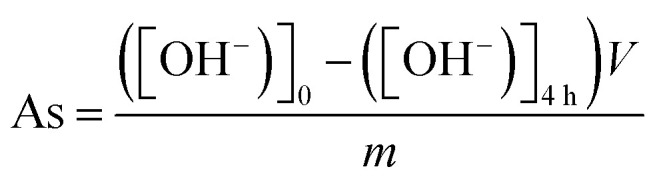
where [OH^−^] is the hydroxide molar concentration determined by the titration (mol dm^−3^), *V* is the volume of NaOH solution added to the studied material (mL) and *m* is the mass of MXenes (mg).

### 2.4. Isomerization of α-pinene

In the studies on the activity of MXene materials in the isomerization of α-pinene, a 10 cm^3^ glass reactor with a reflux condenser and magnetic stirrer with heating were used. The reaction mixture consisted of 1 g of α-pinene and 0.05 g of the appropriate catalyst (catalyst content in the reaction mixture amounted to 5 wt%). The reactor was placed in an oil bath and the reaction mixture was stirred at 500 rpm. The isomerization of α-pinene was performed at the temperature of 160 °C and for the reaction time of 6 h.

For analysis of the composition of post-reaction mixtures, each post-reaction mixture was centrifuged and the post-reaction solution was dissolved in acetone with a 1 : 3 weight ratio. Qualitative analyses of the post-reaction solutions were performed using a GC-MS method with a ThermoQuest apparatus and a DB-5 column (30 m × 0.25 mm × 0.5 μm). The conditions of analyses were as follows: a helium flow rate of 1 mL min^−1^, a sample chamber temperature of 200 °C, and a detector temperature of 250 °C. The furnace temperature was held at 50 °C for 2.5 minutes and then increased to 300 °C at a rate of 10 °C min^−1^.

Quantitative analyses were performed using a Thermo Electron FOCUS chromatograph and a ZB-1701 column (30 m × 0.53 mm × 1 μm). The conditions of analyses were as follows: the helium flow rate of 1 mL min^−1^, the sample chamber temperature of 220 °C, and the detector temperature of 250 °C. The furnace temperature was held at 50 °C for 2 minutes, next it was increased to 100 °C at the rate of 5 °C min^−1^, and finally the temperature was increased to 200 °C at the rate of 10 °C min^−1^.

The composition of the post-reaction solutions was determined using the internal normalization method, and the mass balance was used to calculate the conversion of α-pinene and appropriate product selectivity (camphene, limonene, α-terpinene, γ-terpinene, terpinolene, tricyclene, and *p*-cymene). The summary selectivity of other products, including small amounts of fenchene, polymeric compounds, and oxidation products, was also calculated.

### 2.5. Results and discussion

**Fig. 1:**
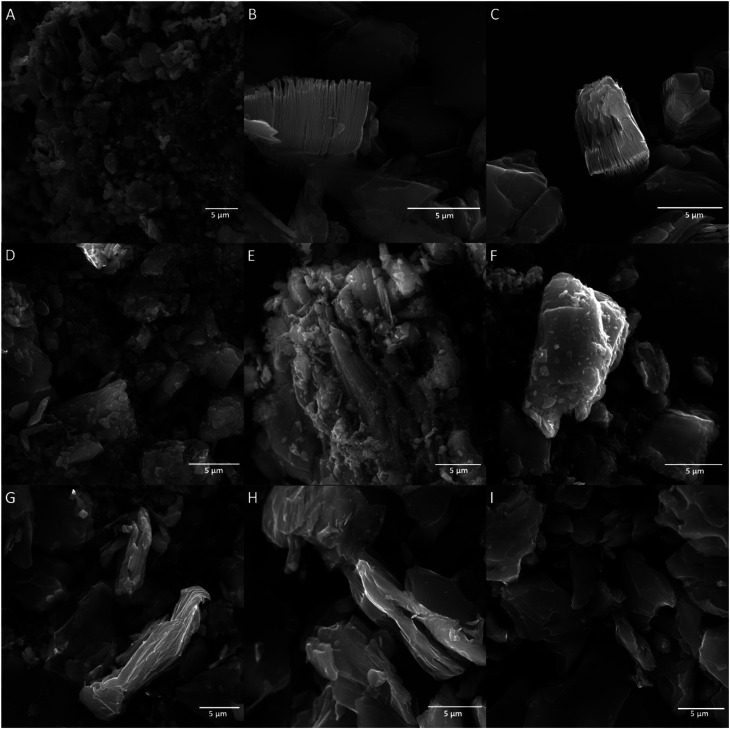
SEM images of MAX phase (A), MXene HF (B and C), MXene HF/H_2_SO_4_ 1 : 3 (D), MXene HF/H_2_SO_4_ 1 : 4 (E), MXene HF/H_2_SO_4_ 1 : 5 (F). MXene HF/HCl 1 : 3 (G), MXene HF/HCl 1 : 4 (H), MXene HF/HCl 1 : 5 (I).

SEM images of MAX phase, MXene HF, MXene HF/H_2_SO_4_*X *: *Y*, and MXene HF/HCl *X* : *Y* are presented in Fig. 1A–I. MAX phase (Fig. 1A) is composed of Ti_3_AlC_2_ particles with irregular shapes. The reference MXene HF (Fig. 1B and C) presents a layered structure with clearly marked spacing between nanosheets. Here, a clear difference between the samples obtained after mixed acids (HF/H_2_SO_4_ and HF/HCl) and HF treatment is observed. MXene HF/H_2_SO_4_*X* : *Y* samples (Fig. 1D–F) do not show layered structures characteristic of MXenes. Their morphology is similar to the MAX phase (Fig. 1A). Whereas, MXene HF/HCl *X* : *Y* (Fig. 1G–I) exhibit a lamellar structure without clearly marked spacing between the nanosheets. It indicates that using a mixture of HF with HCl or H_2_SO_4_ with studied weight ratios for removing Al form Ti_3_AlC_2_ does not cause the creation of accordion-like structure characteristics for MXenes.

**Fig. 2:**
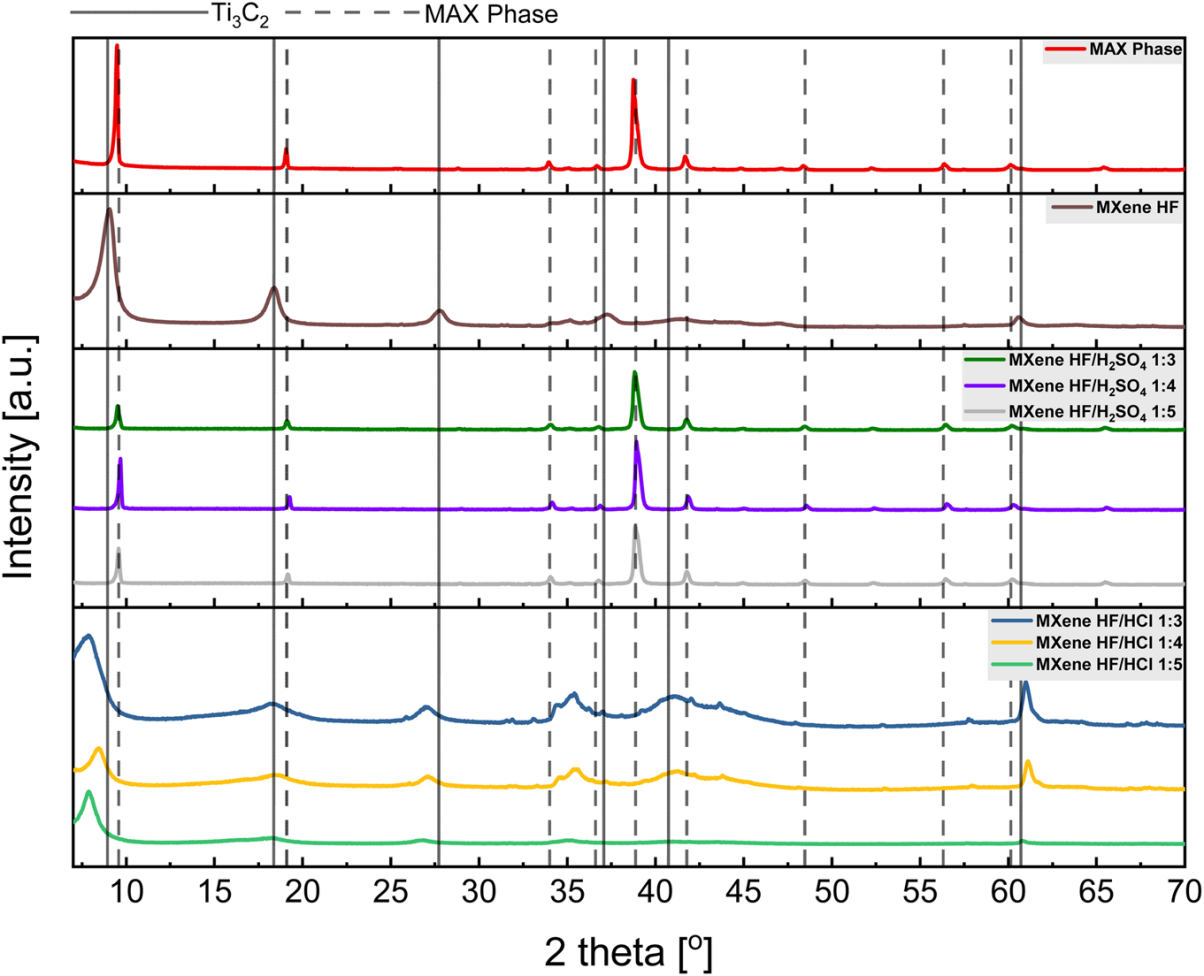
XRD patterns of MAX phase, MXene HF, MXenes HF/H_2_SO_4_ 1 : 3, 1 : 4, 1 : 5 and MXenes HF/HCl 1 : 3, 1 : 4, 1 : 5.

Fig. 2 presents XRD patterns of MAX phase, MXene HF, MXene HF/H_2_SO_4_*X* : *Y*, and MXene HF/HCl *X* : *Y*. The reference MXene HF exhibits reflections at 9.0, 18.2, 27.7, 37.2, 40.7, and 60.7°, typical for Ti_3_C_2_.^30,31^ All MXene HF/H_2_SO_4_*X* : *Y* do not show characteristic reflections of Ti_3_C_2_. Here, peaks located at 9.5, 19.1, 33.9, 36.7, 38.8, 41.7, 48.3, 56.3 and 60.1° related to MAX phase are observed (see pattern of MAX phase). It is also seen that MXene HF/H_2_SO_4_*X* : *Y* exhibit lower intensity peaks than the MAX phase, which indicates that the structure of the MAX phase has been harmed by the reaction with the acids mixture. The materials synthesized *via* removing Al from MAX phase by etching in HF/HCl mixture (MXene HF/HCl *X* : *Y*) show several reflections at 7.7, 18.1, 26.9, 35.4, 40.2, 61.0° characteristics of Ti_3_C_2_ MXene. However, considering MXene HF/HCl *X* : *Y* samples with reference MXene HF, a clear shift of all peaks toward lower angles is detected. It is due to the presence of residual MAX phase in the samples.^32,33^ As the concentration of HF in the HF/HCl mixture decreases, the intensity of peaks derived from Ti_3_C_2_ also decreases, which indicates that the MXenes have not fully developed and give a weaker signal with the same measurement parameters.^34^

**Fig. 3:**
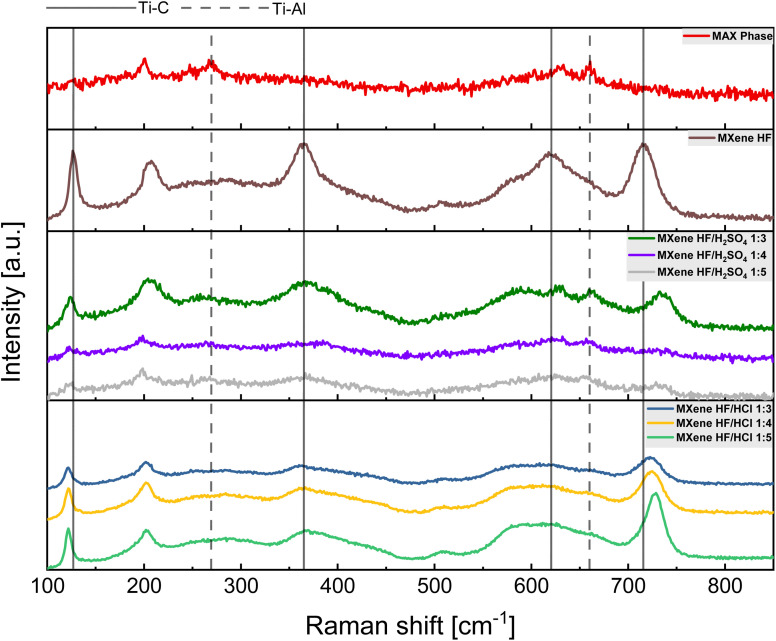
Raman spectra of MAX phase, MXene HF, MXenes HF/H_2_SO_4_ 1 : 3, 1 : 4, 1 : 5 and MXenes HF/HCl 1 : 3, 1 : 4, 1 : 5.

Raman spectra of MAX phase, MXene HF, MXene HF/H_2_SO_4_*X* : *Y* and MXene HF/HCl *X* : *Y* are shown in Fig. 3. The spectrum of MAX phase shows four peaks at ∼201, 269, 631 and 662 cm^−1^, which correspond to Ti–Al vibrations.^35^ MXene HF exhibits five sharp modes located at ∼126, 207, 364, 621 and 716 cm^−1^. The modes observed at ∼126, 364, 621 and 716 cm^−1^ are related to carbon and titanium vibrations. The peak located at ∼207 and ∼716 cm^−1^ indicates proper removal of Al from precursor (MAX phase).^36^ The spectra of MXene HF/H_2_SO_4_*X* : *Y* present modes for both MAX phase and MXene. All MXene HF/H_2_SO_4_*X* : *Y* show four peaks at ∼123, 202, 367, and 733 cm^−1^, as in the case of MXene HF sample. Moreover, the modes located at ∼202 and ∼733 cm^−1^ are the results of the partial removal of the Al from the precursor (MAX phase).^37^ MXene HF/HCl *X* : *Y* exhibit four peaks at ∼121, 201, 363 and 725 cm^−1^, characteristic for Ti_3_C_2_ MXene. Here, it is also seen that all modes have shifted values in respect to MXene HF, which means, not fully formed MXene structure.^38^ The results from Raman are consistent with the results from SEM and XRD.

**Fig. 4:**
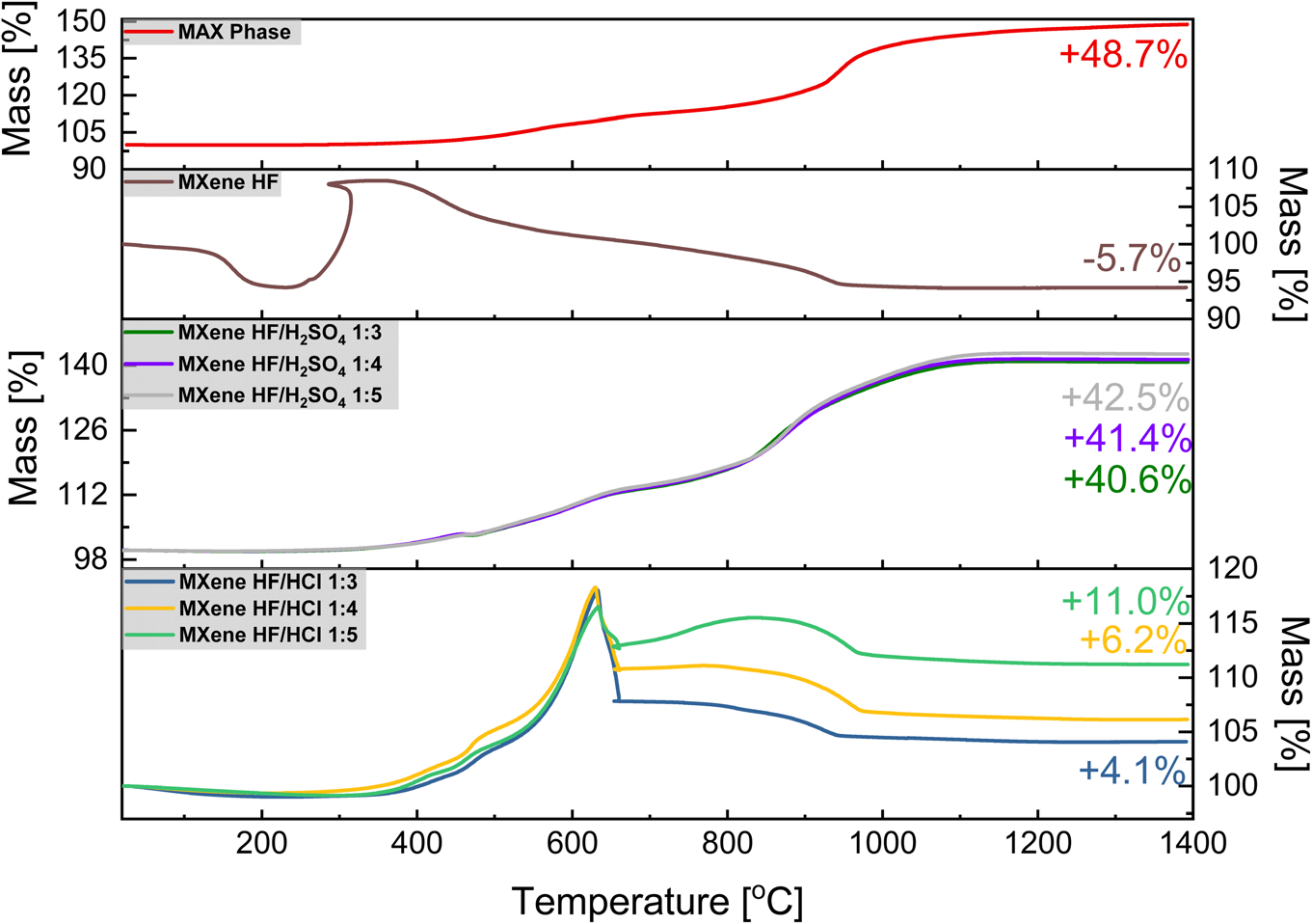
TGA curves of MAX phase, MXene HF, MXenes HF/H_2_SO_4_ 1 : 3, 1 : 4, 1 : 5 and MXenes HF/HCl 1 : 3, 1 : 4, 1 : 5.

**Fig. 5:**
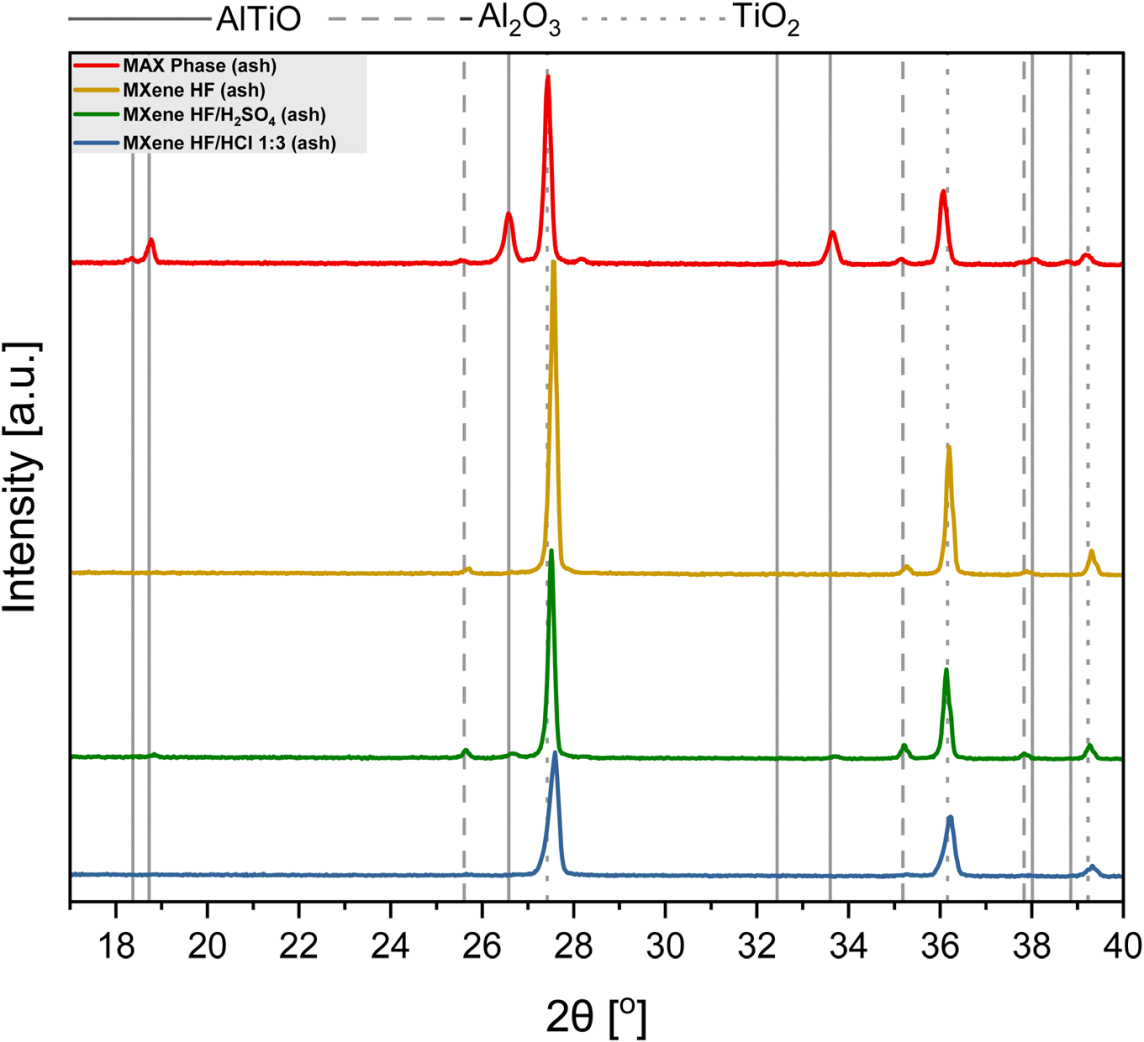
XRD patterns of MAX phase, MXene HF/H_2_SO_4_ 1 : 3 and MXenes HF/HCl 1 : 3 after TGA.

The TGA results of MAX phase, MXene HF, MXene HF/H_2_SO_4_*X* : *Y*, and MXene HF/HCl *X* : *Y* are presented in Fig. 4. For MAX phase the total weight increase of ∼48.7 wt% is observed. Here, two significant steps of mass increase are detected in the TGA curve. The first one (∼307–624 °C; ∼9.1 wt%) represents the oxidation of titanium (formation of TiO_2_), and the second one (∼658–971 °C; ∼27.5 wt%) represents the oxidation of aluminum (formation of Al_2_O_3_). After heating from ∼1000 to 1400 °C aluminum titanium oxide (AlTiO) is formed.^39,40^ MXene HF shows a significant mass increase at ∼310 °C (∼7.4 wt%) followed by a clear mass decrease (∼378–942 °C; ∼12.8 wt%). At ∼1000 °C, the stabilization of the mass of the sample is observed. These mass changes are associated with a parallel process of oxidation of Ti_3_C_2_ and detachment of MXene HF surface functional groups. The total MXene HF mass decreased up to −5.7 wt%, which is in agreement with the literature.^41^ For MXene HF/H_2_SO_4_*X* : *Y* two steps of mass increase, like to MAX phase, are detected. For MXene HF/H_2_SO_4_ 1 : 5, MXene HF/H_2_SO_4_ 1 : 4, and MXene HF/H_2_SO_4_ 1 : 3 the final mass increases are ∼42.5, 41.4, and 40.6 wt%, which is lower by 6.2, 7.3 and 8.1%, respectively, than for MAX phase. MXene HF/HCl *X* : *Y* samples display a significant mass increase, up to ∼624 °C (∼17.9 wt%) manifests oxidation of MXene.^42^ Next, the clear mass loss (up to ∼971 °C) corresponds to the decomposition of MXenes.^41^ The final mass increased by ∼4.1, 6.2, and 11.0 wt% for MXene HF/HCl 1 : 3, MXene HF/HCl 1 : 4, MXene HF/HCl 1 : 5, respectively. Moreover, to identify the product produced after TGA measurement, the ash of MAX phase, MXene HF, MXene HF/HCl 1 : 3 and MXene HF/H_2_SO_4_ 1 : 3 was examined *via* XRD (Fig. 5). The ash obtained after MAX phase thermal treatment is a mixture of AlTiO (18.3, 18.7, 26.6, 28.2, 32.5, 33.6, 38.0, and 38.8°; card no: 01-076-8799), Al_2_O_3_ (25.6, 35.1, and 37.8°; card no: 01-088-4954) and TiO_2_ (27.4, 36.0, and 39.2°; card no: 01-083-3673). The ash of MXene HF exhibits characteristic reflections of TiO_2_. Additionally, a trace amount of Al_2_O_3_ is also detected. XRD pattern of MXene HF/H_2_SO_4_ 1 : 3 after TGA shows reflections corresponding to three phases such as TiO_2_, AlTiO, and Al_2_O_3_. Moreover, it is also seen that the intensity of AlTiO and Al_2_O_3_ reflections is much lower in comparison to that presented in MAX phase after TGA. It means that partial etching of Al from MAX phase occurred in HF/H_2_SO_4_. MXene HF/HCl 1 : 3 after the TGA shows reflections at 27.4, 36.0, and 39.2° corresponding to TiO_2_ (card no.: 01-083-3673). There is also trace amount of Al_2_O_3_ in the ash of MXene HF/HCl 1 : 3.

**Fig. 6:**
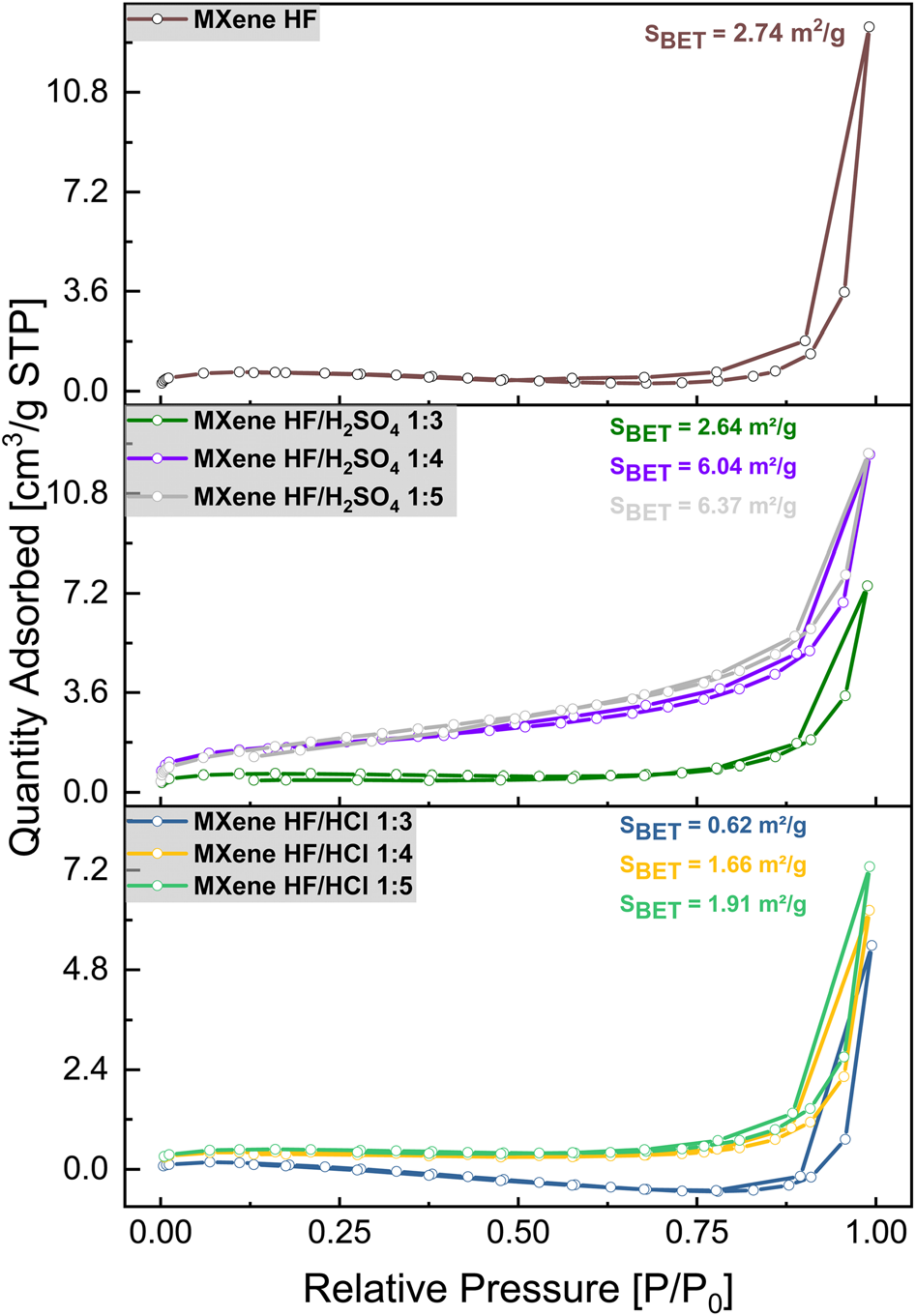
Nitrogen adsorption/desorption isotherms of MXene HF, MXene HF/H_2_SO_4_*X* : *Y* and MXene HF/HCl *X* : *Y*.

The N_2_ adsorption/desorption isotherms of MAX phase, MXene HF, MXene HF/H_2_SO_4_*X* : *Y*, and MXene HF/HCl *X* : *Y* conducted at 77 K are presented in Fig. 6. All studied materials exhibited an IV-type isotherm with H3-type hysteresis loop, indicating multimolecular adsorption.^43^ The specific surface area (SSA) of the samples was calculated using Brunauer–Emmett–Teller (BET) theory (Table 2). The SSA of MXene HF is 2.74 m^2^ g^−1^. For MXene HF/H_2_SO_4_ 1 : 3; 1 : 4 and 1 : 5, SSA is 2.64, 6.04, and 6.37 m^2^ g^−1^, respectively. The SSA of MXene HF/HCl *X* : *Y* is 0.62 (1 : 3), 1.66 (1 : 4) and 1.91 (1 : 5) m^2^ g^−1^. The specific surface area of the materials produced by HF/HCl etching is significantly lower than that obtained after HF and HF/H_2_SO_4_ treatment. MXene HF/H_2_SO_4_ 1 : 5 exhibits the highest BET-SSA (6.37 m^2^ g^−1^) compared to all other samples.

**Table 2:** Acid site concentrations and specific surface area of obtained samples

Sample	Acid sites concentration [mmol g^−1^]	Specific surface area [m^2^ g^−1^]
MXene HF	11.62	2.74
MXene HF/H_2_SO_4_ 1 : 3	2.21	2.64
MXene HF/H_2_SO_4_ 1 : 4	3.05	6.04
MXene HF/H_2_SO_4_ 1 : 5	3.12	6.37
MXene HF/HCl 1 : 3	4.74	0.62
MXene HF/HCl 1 : 4	4.65	1.66
MXene HF/HCl 1 : 5	4.50	1.91

The acid site concentration (As) of MXene HF, MXene HF/H_2_SO_4_*X* : *Y*, and MXene HF/HCl *X* : *Y* is presented in Table 2. The reference MXene HF shows As of 11.62 mmol g^−1^. For both MXene HF/HCl *X* : *Y* and MXene HF/H_2_SO_4_*X* : *Y*. As values are significantly lower in comparison to MXene HF. For the samples produced *via* HF/HCl etching, the value of As is kept in the following order: MXene HF/HCl 1 : 3 (4.74 mmol g^−1^) > MXene HF/HCl 1 : 4 (4.65 mmol g^−1^) > MXene HF/HCl 1 : 5 (4.50 mmol g^−1^). It means that the higher HF content in the HF/HCl mixture the higher concentration of acid sites. The acid site concentration for MXene HF/H_2_SO_4_ 1 : 3, 1 : 4 and 1 : 5 was 2.21, 3.05 and 3.12 mmol g^−1^, respectively. Here, an opposite correlation for MXene HF/HCl *X* : *Y* was observed, along with the lower content of HF in HF/H_2_SO_4_ mixture, the higher concentration of acid sites is detected.

**Fig. 7:**
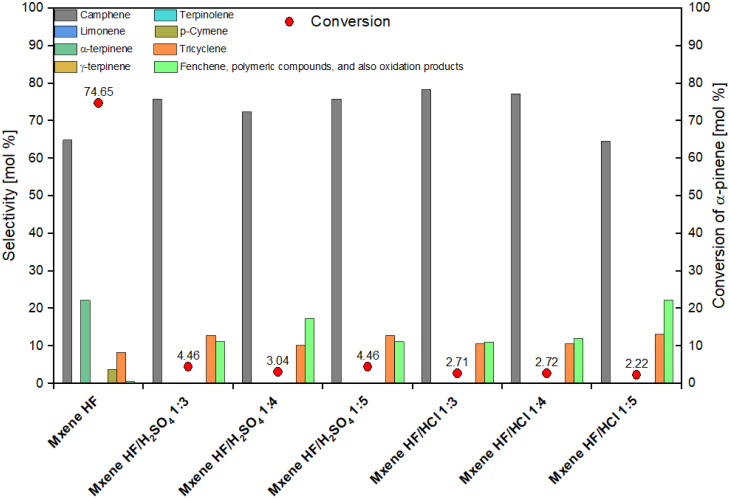
Results of the catalytic tests for the isomerization of α-pinene for MXene HF, MXene HF/H_2_SO_4_*X* : *Y*, and MXene HF/HCl *X* : *Y*.

In the next stage of the studies, the catalytic activity of MXene HF, MXene HF/H_2_SO_4_*X* : *Y*, and MXene HF/HCl *X* : *Y* in the reaction of isomerization of α-pinene was examined. The obtained results are presented in Fig. 7.

It is seen from Fig. 7 that taking into account the value of conversion of α-pinene the highest activity showed MXene HF (conversion of α-pinene amounted to 74.65 mol%). All MXene HF/HCl *X* : *Y* and MXene HF/H_2_SO_4_*X* : *Y* showed a significant, approximately 28-fold, decrease in activity. At the same time, the samples of MXene HF/H_2_SO_4_*X* : *Y* were characterized by a slightly higher activity than the materials of MXene HF/HCl *X* : *Y*. The conversion of α-pinene is 4,46, 3.04 and 4.46 mol% for MXene HF/H_2_SO_4_ 1 : 3, MXene HF/H_2_SO_4_ 1 : 4, and MXene HF/H_2_SO_4_ 1 : 5, respectively. On the other hand, the increase of about 8–10 mol% in the selectivity of transformation of α-pinene to camphene and also the increase of about 2–4 mol% in the selectivity of transformation to tricyclene was detected for the samples synthesized by the treatment with the mixtures of 2 acids. In particular, the increase in selectivity of the transformation to the first of these compounds is advantageous as camphene is the preferred product of this reaction. It should also be noted that only in the isomerization of α-pinene performed on MXene HF, α-terpinene (selectivity 22.27 mol%) and *p*-cymene (selectivity 3.88 mol%) were formed. Moreover, a significant, 20 to 40-fold increase in the selectivity of α-pinene transformation to other products was observed on catalysts which were obtained by the treatment with the mixtures of 2 acids. Other products can be such products as fenchene, polymeric compounds, and also oxidation products. The highest increase in the amount of these compounds was noted for the MXene HF/HCl 1 : 5 (more than 40 times). It is also visible that the lowest selectivity of transformation to camphene was simultaneously obtained on this sample (64.62 mol%) – this is the value comparable to the value obtained on the MXene HF.

The above-mentioned observations can be explained by taking into account the structure of MXene HF sample and the samples of catalysts obtained by the treatment with the mixture of 2 acids. For MXene HF, which is characterized by the accordion-like structure with clearly marked spacing between layers, the formation of bicyclic products: camphene and tricyclene, and monocyclic products: α-terpinene and *p*-cymene, was characteristic. A slight selectivity of the transformation to “other products” (fenchene, polymeric compounds, and also oxidation products) was also observed for MXene HF. In the case of the catalysts obtained by the treatment with the mixture of 2 acids, we observe the formation of only bicyclic products and the increase in the amount of products such as fenchene, polymeric compounds, and also oxidation products. These products are characterized by much larger molecules (often very spatially expanded), requiring more space for reactions involving them to occur. Looking at the structure of these catalysts (MXene HF/H_2_SO_4_*X* : *Y* samples do not show layered structure and MXene HF/HCl *X* : *Y* samples exhibit the lamellar structure without clearly marked spacing between the nanosheets) and the MXene HF catalyst structure, it can be assumed that in the case of the MXene HF, the formation of monocyclic products was related to the diffusion of α-pinene molecules into narrow spaces between the layers of this catalyst. Probably the diffusion of α-pinene molecules was difficult and only some of the α-pinene molecules reached there. It should also be taken into account that limitations in the diffusion to the area between the layers may also be related to the presence of anions in these areas (*e.g.* Cl^−^ and F^−^) that were not completely washed out from the catalyst after synthesis. These anions may additionally limit the diffusion of α-pinene molecules to the area between the layers and block their access to the active sites. Due to the small space between the layers, the formation of monocyclic products and their further transformations (formation of *p*-cymene) was favored. However, the remaining molecules of α-pinene remained on the surface of the layered catalyst particles and the α-pinene transformation process probably took place on them. Due to much smaller steric constraints on the surface of large, layered MXene HF catalyst particles, it was possible to obtain large, bicyclic products such as camphene and tricyclene. In the case of the MXene HF/H_2_SO_4_*X* : *Y* and MXene HF/HCl *X* : *Y* catalysts the lack of spatial limitations related to the size of the reaction space (as was for reactions taking place between layers) made it possible to obtain bicyclic products and, at the same time, steric factors did not force the formation of monocyclic products. Also, the formation of fenchene, polymeric products, and oxidation products with higher selectivity is associated with lower steric restrictions on the surface of the catalyst particles. Moreover, due to the significantly reduced number of acid centers on the surface of particles of MXene HF/H_2_SO_4_*X* : *Y* and MXene HF/HCl *X* : *Y* catalysts, the conversion of α-pinene was significantly reduced. MXene HF was characterized by the concentration of acid sites amounted to 11.62 mmol g^−1^, but for MXene HF/HCl *X* : *Y* and MXene HF/H_2_SO_4_*X* : *Y*, the concentration of acid sites was about 2.5–5.5 times smaller (see Table 2). At the same time, it can also be noted that although the MXene HF/HCl *X* : *Y* samples were characterized by a slightly higher concentration of acid sites, their activity in the isomerization of α-pinene was lower than the MXene HF/H_2_SO_4_*X* : *Y* materials (taking into account the conversion of α-pinene), which were characterized by almost 2 times greater specific surface area. This difference in the activity of samples obtained by the treatment with the mixtures of two acids may therefore probably be due to the different availability of acid sites on the particles of these materials. Probably in MXene HF/H_2_SO_4_*X* : *Y* acid sites are more accessible.

**Fig. 8:**
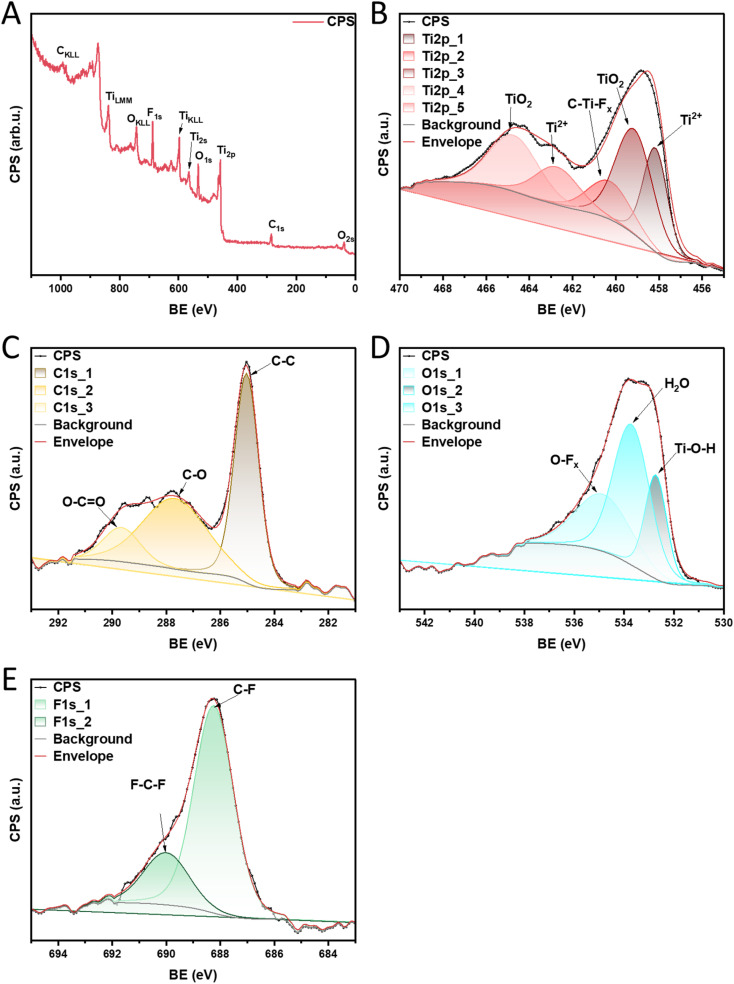
XPS survey spectra of MXene HF (A), high-resolution XPS of elemental: Ti 2p (B), C 1s (C), O 1s (D), and F 1s (E).

To clarify what functional groups may constitute the active centers on which the α-pinene isomerization reaction takes place, XPS analysis was performed for the material with the highest catalytic activity (MXene HF). The sample overview is presented in Fig. 8A. The results confirmed the presence of Ti 2p, C 1s, O 1s, and F 1s on the surface of the MXene HF. The detailed XPS spectra of Ti 2p (Fig. 8B) demonstrate characteristic peaks at ∼458.1, 459.0, 460.4, 462.8, and 464.8 eV which is assigned to Ti^2+^, Ti–O, C–Ti–F_*x*_, Ti^2+^, and Ti–O, respectively.^44^ High-resolution XPS C 1s spectrum is shown in Fig. 8C. MXene HF exhibits an intense peak located at ∼285.0 eV, which is attributed to the C–C bonds. The material shows two additional peaks at ∼287.7 and 289.7 eV, which correspond to C–O and O–C=O bonds, respectively.^44^ As depicted in Fig. 8D the spectrum for O 1s is deconvoluted into three components corresponding to the presence of different oxygen species at the surface of MXene HF. The first peak at ∼532.0 eV is ascribed to Ti–O–H bonding. The second peak at ∼533.8 eV is due to adsorbed H_2_O. The third peak is located at ∼535.0 eV and corresponds to O–F_*x*_ bonding.^45,46^ As depicted in Fig. 8E F 1s region is deconvoluted into 2 peaks at ∼688.3, and 690.0 eV, which is attributed to C–F, and F–C–F, respectively.^47^

The results of tests carried out using the XPS method for the MXene HF catalyst confirmed the presence of Ti–OH groups on the surface of this material, which constitute the active centers of the catalyst (proton source for the α-pinene isomerization reaction). They may, among others, be formed from titanyl groups (Ti=O) in the hydrolysis process. Similarly, C–OH groups present on the surface of the tested catalyst may be the proton source for the isomerization reaction. The C–Ti–F_*x*_, O–F_*x*_, C–F and F–C–F groups also attract attention. They indicate the binding of fluorine in the structure of the tested material, but at the same time, the formation of such groups indicates that protons from HF must also have been bound in some way in the structure. It can be assumed, for example, that HF reacting with the carbonyl group (C=O group) causes the formation of CF(OH) groups, in which the OH group can be the proton source for the isomerization reaction. Similarly, the titanyl group (Ti=O) reacts with HF to form TiF(OH) groups, and here also the OH group can be the proton source for the isomerization reaction. However, a detailed explanation of the mechanism of HF reaction with surface groups and its participation in the formation of acidic centers, which can be the source of a proton for the isomerization reaction, requires further, very detailed research in the future.

## 3. Conclusion

Ti_3_C_2_ MXenes-based materials were synthesized and applied as catalysts in the reaction of α-pinene isomerization. The impact of the etching medium (HF; HF/HCl and HF/H_2_SO_4_: weight ratios: 1 : 3, 1 : 4, and 1 : 5) on the composition, morphology, and structure of the produced samples was investigated. During the catalytic tests, the highest activity showed MXene HF, the sample with layered structure and clearly marked spacing between nanosheets, which was obtained by the treatment of MAX phase only with HF (conversion of α-pinene achieved a value of 74.65 mol%). The catalytic tests also showed that materials produced by the treatment of MAX phase with HF/H_2_SO_4_ mixtures were slightly more active than the samples obtained by HF/HCl treatment. The latter materials were characterized by the lower concentration of acid sites, but probably these acid sites were more accessible to α-pinene molecules. In general, it can be said that using HF/H_2_SO_4_ and HF/HCl mixtures for removing Al form MAX phase led to a significant reduction in the conversion of α-pinene (on average about 28-fold), while increasing by about 10 mol% in the selectivity of the transformation to the most desirable product – camphene. To summarize, further modifications of the method of aluminum etching from MAX phase should go towards such a selection of compounds used for etching and their mutual ratio that, while maintaining the increased selectivity of transformation to camphene, significantly increase the conversion of α-pinene.

## Conflicts of interest

There are no conflicts to declare.

## Supplementary Material

## References

[cit1] Lim K., Garrick R. (2022). *et al.*, Fundamentals of MXene synthesis. Nat. Synth..

[cit2] Li X. (2022). *et al.*, MXene chemistry, electrochemistry and energy storage applications. Nat. Rev. Chem.

[cit3] Salehi B., Upadhyay S., Orhan I. E., Jugran A. K., Jayaweera Sumali L. D., Dias D. A., Sharopov F., Taheri Y., Martins N., Baghalpour N., William Cho C., Sharifi-Rad J. (2019). Therapeutic Potential of α- and β-Pinene: A Miracle Gift of Nature. Biomolecules.

[cit4] Allenspach M., Steuer C. (2021). α-Pinene: A never-ending story. Phytochemistry.

[cit5] Nyamwihura R. J., Ogungbe I. V. (2022). The pinene scaffold: its occurrence, chemistry, synthetic utility, and pharmacological importance. RSC Adv..

[cit6] Coelhan M., Maurer M. (2005). Synthesis of Two Major Toxaphene Components and Their Photostabilities. J. Agric. Food Chem..

[cit7] Glaser C. , US Pat.US875062 A, Maryland, 31 Dec, 1907

[cit8] Bintang L., Jinquan Y., Aiqun E., Ping Z., Shude X. (1995). Study on selective alkylation of guaiacol with camphene over H-Mordenite. Chin. J. Org. Chem..

[cit9] Girola N., Figueiredo C. R., Farias C. F., Azevedo R. A., Ferreira A. K., Teixeira S. F., Capello T. M., Martins E. G. A., Matsuo A. L., Travassos L. R., Lago J. H. G. (2015). Camphene isolated from essential oil of *Piper cernuum* (Piperaceae) induces intrinsic apoptosis in melanoma cells and displays antitumor activity *in vivo*. Biochem. Biophys. Res. Commun..

[cit10] Król S. K., Skalicka-Wożniak K., Kandefer-Szerszeń M., Stepulak A. (2013). The biological and pharmacological activity of essential oils in the treatment and prevention of infectious diseases. Postepy Hig. Med. Dosw..

[cit11] Martın-Luengoa M. A., Yatesb M., Martınez Domingo M. J., Casala B., Iglesiasa M., Estebana M., Ruiz-Hitzky E. (2008). Synthesis of p-cymene from limonene, a renewable feedstock. Appl. Catal. B.

[cit12] Royals E. E., Horne Jr S. E. (1951). Conversion of d-Limonene to l-Carvone. J. Am. Chem. Soc..

[cit13] Arrieta M. P., Lopez J., Ferrandiz S., Peltzer M. A. (2013). Characterization of PLA-limonene blends for food packaging applications. Polym. Test..

[cit14] Pybus D. and Sell C., The Chemistry of Fragrances, The Royal Society of Chemistry, 1999, pp. 24–51

[cit15] Gastao E. ,US Pat.US 2551795 A, Du Pont, 8 May 1951, 10.1039/9781847552044-00024

[cit16] Arata K., Tanabe K. (1979). Isomerization of α-plnene oxide over solid acids and bases. Chem. Lett..

[cit17] Sánchez-Velandia J. E. (2021). *et al.*, Selective synthesis of camphene from isomerization of α- and β-pinene over heterogeneous catalysts. Microporous Mesoporous Mater..

[cit18] Launay F., Jarry B., Bonardet J. L. (2009). Catalytic activity of mesoporous Ga-SBA-15 materials in α-pinene isomerisation: Similarities and differences with Al-SBA-15 analogues. Appl. Catal. A: General.

[cit19] Wu C., Liu H., Zhuang C., Du G. (2014). Study on Mesoporous PW/SBA-15 for Isomerization of α-Pinene. Appl. Mech. Mater..

[cit20] Luque R., Campelo J. M., Conesa T. D., Luna D., Marinad J. M., Romero A. A. (2007). Ga-MCM-41 synthesis and catalytic activity in the liquid-phase isomerisation of α-pinene. Microporous Mesoporous Mater..

[cit21] Ecormier M. A., Lee A. F., Wilson K. (2005). High activity, templated mesoporous SO4/ZrO2/HMS catalysts with controlled acid site density for α-pinene isomerisation. Microporous Mesoporous Mater..

[cit22] Rachwalik R., Olejniczak Z., Jiao J., Huang J., Hunger M., Sulikowski B. (2007). Isomerization of α-pinene over dealuminated ferrierite-type zeolites. J. Catal..

[cit23] Unveren E., Gunduz G., Cakicioglu-ozkan F. (2005). Isomerization of Alpha-pinene Over Acid Treated Natural Zeolite. Chem. Eng. Commun..

[cit24] Wijayati N., Pranowo H. D., Jumina J., Triyono T., Chuah G. H. (2013). Characterization of ZHY and TCA/ZHY Catalysts for Hydration of α-Pinene. Int. J. Chem. Eng. Appl..

[cit25] Akpolat O., Gunduz G., Ozkan F., Besug N. (2005). General, Isomerization of α-pinene over calcined natural zeolites. Appl. Catal. A.

[cit26] Gynduz G., Murzin D. Y. (2002). Influence of catalyst pretreatment on α-pinene isomerization over natural calys. React. Kinet. Catal. Lett..

[cit27] Miądlicki P., Wróblewska A., Kiełbasa K., Koren Z. C., Michalkiewicz B. (2021). Sulfuric acid modified clinoptilolite as a solid green catalyst for solvent-free α-pinene isomerization process. Microporous Mesoporous Mater..

[cit28] Zielińska B. (2020). *et al.*, High catalytic performance of 2D Ti3C2Tx MXene in α-pinene isomerization to camphene. Appl. Catal., A.

[cit29] Vilcocq L., Spinola V., Moniz P., Duarte L. C., Carvalheiro F., Fernandes C., Castilho P. (2015). Catal. Sci. Technol..

[cit30] Tariq A., Irfan Ali S., Akinwande D., Rizwan S. (2018). Efficient visible-light photocatalysis of 2D-MXene nanohybrids with Gd3+-and Sn4+-codoped bismuth ferrite. ACS omega.

[cit31] Shuck C. E. (2020). *et al.*, Scalable synthesis of Ti3C2Tx mxene. Adv. Eng. Mater..

[cit32] Mahmood M. (2021). *et al.*, Synthesis of ultrathin MnO2 nanowire-intercalated 2D-MXenes for high-performance hybrid supercapacitors. Energy Fuels.

[cit33] Anayee M. (2022). *et al.*, Kinetics of Ti3AlC2 Etching for Ti3C2T x MXene Synthesis. Chem. Mater..

[cit34] Limbu T. B. (2020). *et al.*, Green synthesis of reduced Ti 3 C 2 T x MXene nanosheets with enhanced conductivity, oxidation stability, and SERS activity. J. Mater. Chem. C.

[cit35] Liu F. (2016). *et al.*, Preparation and methane adsorption of two-dimensional carbide Ti 2 C. Adsorption.

[cit36] Limbu T. B. (2020). *et al.*, Green synthesis of reduced Ti 3 C 2 T x MXene nanosheets with enhanced conductivity, oxidation stability, and SERS activity. Journal of Materials Chemistry C.

[cit37] Liu F. (2016). *et al.*, Preparation and methane adsorption of two-dimensional carbide Ti2C. Adsorption.

[cit38] Li J., Wang H., Xiao X. (2020). Intercalation in two-dimensional transition metal carbides and nitrides (MXenes) toward electrochemical capacitor and beyond. Energy Environ. Mater..

[cit39] Haftani M. (2016). *et al.*, Studying the
oxidation of Ti2AlC MAX phase in atmosphere: A review. Int. J. Refract. Hard Met..

[cit40] Ostroman I. (2023). *et al.*, Highly Reversible Ti/Sn Oxide Nanocomposite Electrodes for Lithium Ion Batteries Obtained by Oxidation of Ti3Al (1-x) SnxC2 Phases. Small Methods.

[cit41] Simonenko E. P. (2023). *et al.*, Gas-Sensitive Properties of ZnO/Ti2CTx Nanocomposites. Micromachines.

[cit42] Wang X. H., Zhou Y. C. (2002). Oxidation behavior of Ti 3 AlC 2 powders in flowing air. J. Mater. Chem..

[cit43] Wen Y. (2019). *et al.*, FeNC/MXene hybrid nanosheet as an efficient electrocatalyst for oxygen reduction reaction. RSC adv..

[cit44] Wan S. (2020). *et al.*, Strong sequentially bridged MXene sheets. Proc. Natl. Acad. Sci..

[cit45] Yang W. (2020). *et al.*, Covalently sandwiching MXene by conjugated microporous polymers with excellent stability for supercapacitors. Small Methods.

[cit46] Shah S. A. (2017). *et al.*, Template-free 3D titanium carbide (Ti 3 C 2 T x) MXene particles crumpled by capillary forces. Chem. Commun..

[cit47] Chen G., Zhang J., Yang S. (2008). Fabrication of hydrophobic fluorinated amorphous carbon thin films by an electrochemical route. Electrochem. Commun..

